# Accuracy Analysis of a Dam Model from Drone Surveys

**DOI:** 10.3390/s17081777

**Published:** 2017-08-03

**Authors:** Elena Ridolfi, Giulia Buffi, Sara Venturi, Piergiorgio Manciola

**Affiliations:** DICA Department of Civil and Environmental Engineering, University of Perugia, 06125 Perugia, Italy; giulia.buffi@unipg.it (G.B.); sarvent@gmail.com (S.V.); piergiorgio.manciola@unipg.it (P.M.)

**Keywords:** dam survey, monitoring, UAV, ground control point, marker optimization, dense point cloud, vulnerability analysis, accuracy

## Abstract

This paper investigates the accuracy of models obtained by drone surveys. To this end, this work analyzes how the placement of ground control points (GCPs) used to georeference the dense point cloud of a dam affects the resulting three-dimensional (3D) model. Images of a double arch masonry dam upstream face are acquired from drone survey and used to build the 3D model of the dam for vulnerability analysis purposes. However, there still remained the issue of understanding the real impact of a correct GCPs location choice to properly georeference the images and thus, the model. To this end, a high number of GCPs configurations were investigated, building a series of dense point clouds. The accuracy of these resulting dense clouds was estimated comparing the coordinates of check points extracted from the model and their true coordinates measured via traditional topography. The paper aims at providing information about the optimal choice of GCPs placement not only for dams but also for all surveys of high-rise structures. The knowledge a priori of the effect of the GCPs number and location on the model accuracy can increase survey reliability and accuracy and speed up the survey set-up operations.

## 1. Introduction

In recent years, dam safety has acquired increasing attention because of the high number of accidents and failures that have occurred. For instance, in the United States from 2005 to 2013 the State Dam Safety Program reported 173 dam failures and 587 occurrences that, without intervention, would likely have resulted in dam failure [1]. The typical causes of failures are foundation deterioration, including uneven settlement and earthquakes [2]; overtopping as a result of inadequate spillway crests [3], debris blockage of spillways and settlement of the dam crest [4]; piping, including high pore pressure and embankment slips [5] and others, such as improper construction, defective materials and also acts of warfare [6]. The need for sharing experiences related to this issue led to the establishment of the International Commission on Large Dams (ICOLD) whose mission is the dissemination of information to increase designers’ and managers’ awareness of the events which can lead to a disaster [7,8]. 

In this framework, monitoring operations and vulnerability assessment of dams plays a crucial role in preventing catastrophes and thus safeguarding human lives. Because of the large dimensions and the low accessibility of dams, a unique opportunity for monitoring is offered by Unmanned Aerial Vehicle (UAV) systems. Indeed, UAVs are extremely advantageous for visual investigation of large-scale structures such as dams and retention walls [9]. The use of UAVs has acquired a key role because of their fast and low cost operations and of the possibility of reaching places that are otherwise difficult or impossible to access directly [10,11,12]. UAV surveys find applications in several fields, for instance UAVs are used for vegetation monitoring [13], precision agriculture enhancement [14], and information acquisition concerning damages caused by natural disasters such as earthquakes [15,16]. In hydrology, drones are used to measure open channel surface velocities [17,18]. 

As structures are subject to deterioration due to increasing loads, weather conditions and ageing processes, drones offer a unique opportunity. As a matter of fact, conventional inspections are based on visual investigations which are time consuming, require technical experts and are therefore expensive. In this framework, drones provide an important contribution to strategies for monitoring of structures. For instance, Hallermann et al. [19] presented a methodology for inspecting ageing structures combining conventional inspection measurements with modern photogrammetric computer vision methods for geo-referenced structures, 3D-modeling and automatic post-flight damage detection. Achille et al. [20] made use of drones to survey vertical structures after an earthquake occurred in Mantua (IT). Grenzdörffer et al. [21] coupled terrestrial laser scanner (TLS) measurements with drone photogrammetry to detail a cultural monument in Germany. Hallermann and Morgenthal [22] and Hallermann et al. [23] used a drone to inspect bridges and viaducts. 

In this framework, the use of the UAV technique for inspection and geometry recreation of dams is highly appropriate [24] and it can substantially improve monitoring and survey operations. Data acquired during the survey can be reliably considered as the basis for the construction of a 3D model for vulnerability studies. Indeed, a dam is a multi-hazard vulnerable structure that can be severely affected by earthquakes, flooding and terrain stability-related issues. The 3D model of the dam can be utilized to build a finite element (FE) model and perform static and dynamic analyses of the structure. Moreover, many authors have dealt with the simulation of dam breaks in a 3D hydraulic framework to better represent the vertical acceleration influencing the flow [25,26,27,28,29,30]. Thus, the availability of a 3D model of the dam is of great interest for providing a proper representation of the breaking event. Among the other applications of a 3D dam model, we can also list the 3D model of spillways to investigate the dynamics of the flow that spills over [31,32].

The 3D model of an object is built up from the images acquired during the UAV survey. Indeed, thanks to the Structure from Motion (SfM) technique, it is possible to build a dense point cloud (i.e., a 3D model) of an object from the 2D images acquired during a drone survey [33]. 

The SfM technique revolutionized three-dimensional topographic surveys in many fields, such as physical geography, by opening data collection and processing to a wider public [34]. Indeed, the SfM is a cheap technique that does not need specialized supervision. These two characteristics played the main role in the dissemination of SfM as highlighted by Micheletti et al. [35] who illustrated the potential of SfM applications in geomorphological research. Micheletti et al. [36] investigated the potential of freely available SfM tools to process high-resolution topographic and terrain data acquired through a smartphone. Among the other applications, SfM is used to investigate wave run-up [37], to determine soil erosion [38,39], to map landslides and to assess glacier movement [40,41]. Many authors have coupled the SfM technique with drones for surveying and modeling structures. Indeed, thanks to drones it is possible to acquire images of entire buildings including the roof and other parts inaccessible to the scanner [42]. For instance, Bolognesi et al. [43] detailed the “Delizia del Verginese” Castle in central Italy; Koutsoudis et al. [44] reconstructed an Ottoman monument located in Greece, estimating the accuracy of the data produced by a multi-image 3D reconstruction technique in terms of surface deviation and distance measurement accuracy. Among the other benefits, 3D modeling of cultural heritage sites has led to scientific and cost-effective improvements in documenting and archiving operations [45]. Westoby et al. [46] applied the SfM technique to a breached moraine-dam; Hallermann et al. [19] obtained a high-resolution orthophoto of a dam located in Germany to inspect its surface and to identify damages. Reagan et al. [47] combined autonomous flight with 3D digital image correlation to inspect bridges.

To georeference the dense point cloud and thus obtain an in-scale model of a specific object, the coordinates of a specific number of points (i.e., ground control points; GCPs) deployed on the object are used. The coordinates of GCPs are estimated by traditional survey techniques acquiring the coordinates of objects named markers. Markers are usually square shaped objects deployed on the dam surface before the survey. Subsequently, to determine the accuracy of the 3D model, it is necessary to compare the coordinates of check points (i.e., CPs) lying on the dense point cloud with their actual coordinates, acquired by traditional topographic methods. Therefore, the accuracy of the 3D model is strongly influenced by the location and number of GCPs used to georeference the model itself. 

In literature, some authors have dealt with the effects of several features on the dense point cloud accuracy, such as the flight altitude, the inclination of the camera’s optimal axis, the image network geometry, image matching performance, surface texture and lighting conditions, GCPs number, positioning and accuracy [48]. For instance, Bolognesi et al. [43] investigated the accuracy of the dense point cloud of a historic castle by varying the flight altitude, the camera optical axis inclination and the number and location of GCPs. Barry and Coakley [49] estimated the accuracy achievable using a UAV on a 2-hectar site varying the number of GCPs. Tahar [50] evaluated the effect of different number of GCPs on the photogrammetric survey on a hilly area in Malaysia, while Tahar et al. [51] assessed the effect of position and number of GCPs on DEM generation. Mesas-Carrascosa et al. [52,53] analyzed the effect of three flight altitudes, flight modes (stop and cruising modes) and ground control point settings on ortho-mosaicked images. According to literature guidelines, GCPs should be widely distributed across the target area [54] and at the edge or outside [55] to enclose the area of interest [56,57,58,59]. According to Harwin and Lucieer [60], GCPs distribution needs to be adapted to the surveyed object and to the distance of the UAV. GCPs should be placed between 1/5 and 1/10 of the distance from the UAV to the surveyed object. Work investigating the effect of marker configurations on the dense point cloud of a high-rise building and, more specifically, of a dam is still to be done.

The placement of control points, especially in giant structures such as dams, is a time and money-consuming task. Moreover, some dam portions can be reached during specific seasons only because of the variable hydrostatic level in the reservoir. In this framework, the knowledge a priori of the effect of GCPs location choice on the accuracy of the dense cloud is relevant information that could not only increase the 3D model reliability but also speed up the experimental set up and thus, the dam survey itself. 

This work focuses on the specific issue of marker deployment, while the other features are kept constant. This allows us to investigate this issue in detail. Given that no study on the effects of GCPs deployment on a masonry dam yet exists, the original contribution of this paper is the investigation of the accuracy affecting the 3D model of a dam obtained by a drone survey exploring different GCPs layouts. Results obtained by this study can find application for the improvement of large building surveys.

As the connection between the dam and the terrain plays a key role in ensuring the stability of the dam itself, the survey needs to be as accurate as possible to correctly represent and monitor the interconnection between dam and terrain. Thus, a second original contribution is represented by the investigation of the GCPs location effect on the dam’s boundaries (i.e., the abutments). Moreover, the 3D model accuracy is highly affected by the presence of singularities, such as the spillways. Indeed, a proper location of GCPs needs to take into account the presence of openings in order to obtain an accurate dense point cloud.

This paper investigates the case study of the Ridracoli dam (IT); an arch dam located in central Italy constructed principally for drinkable water supply purposes. An accurate assessment of the vulnerability of this structure is of utmost importance to ensure the continuous availability of the water resource from its multipurpose retention basin [61]. It is a masonry arch-shaped dam with a height of 103.5 m and crowning length equal to 32 m. The dam widths at the foundations and at the crowning are equal to 30 m and 7 m, respectively. For this type of structure, the mean vertical error can be assumed acceptable if equal to or lower than the 0.1% of the dam height. In fact, this value is consistent with finite element analysis [62] and hydraulic modeling [63]. The analysis is performed on the upstream face of the dam generating a unique dense point cloud of this portion. The upstream face was chosen for three reasons: (1) markers have a higher concentration and are more uniformly spaced on the upstream face than on the rest of the structure; (2) it offers the possibility of better investigating the spillway openings and the interconnection terrain-structure; (3) it highlights the similarity between the upstream face and other high-rise buildings. In fact, this paper aims to provide useful guidelines on marker location that could be valid also for other high-rise constructions. It is worth underlining that the downstream face of the dam is inaccessible, therefore it was not possible to deploy markers on it.

The paper is organized as follows: first, the case study area is presented and details about the experimental set up are provided. Several configurations of markers are presented and the survey technique to estimate marker coordinates is presented. Then, the drone survey is reported. Secondly, we detail the dense point cloud construction by varying GCPs layout. The errors between CPs coordinates on the dam model and on the actual dam are estimated and analyzed for each layout. In the results and discussion section we summarize our findings, focusing on the effects of error on dam boundaries.

## 2. Case Study and Experimental Set-Up Description

**Figure 1:**
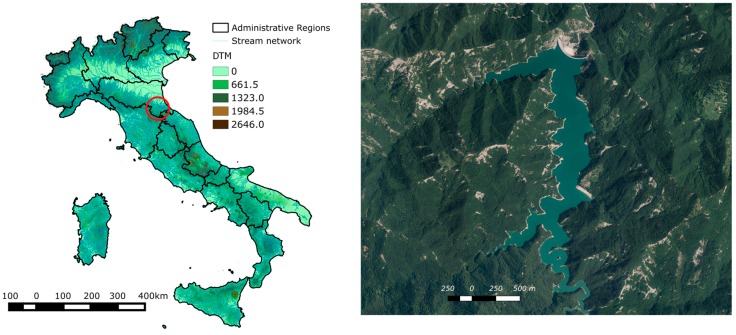
DTM of Italy (left panel), the red circle indicates the case study area, while the dam case study i.e., the Ridracoli dam and the Ridracoli lake are on the right panel.

**Figure 2:**
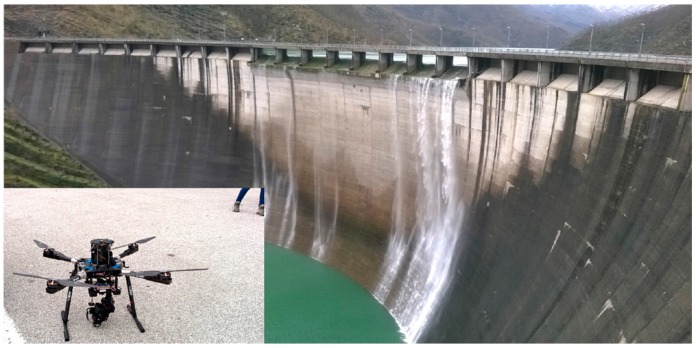
Ridracoli dam while overflowing. Small panel: HIGHONE 4HSEPRO quadrotor mounting a gimbal system and a SONY Alpha 7R while at rest.

The markers layout assessment is performed using the Ridracoli dam as a case study. The dam was named after the homonymous lake and it was built on the Bidente River in the province of Forlì –Cesena (central Italy), generating a basin of about 33 million m^3^ of water (Figure 1). The dam supplies water to 48 municipalities in provinces throughout central Italy and, since 1989, to the Republic of San Marino [64]. The Ridracoli dam has a double-curvature arch-gravity structure with a maximum height of 103.5 m and a crest length of 432 m at 561 m a.s.l. The structure is divided into 27 independent parts called ashlars that avoid cracks that can result from hydrostatic and thermal loads. Vertical contraction joints ensure the continuity of the structure. Eight free spillway gates, located in the centre of the dam crown, allow the overflow of the dam (Figure 2). 

In the following sub-sections the drone survey and the topographical technique to acquire marker coordinates will be detailed. The materialization of targets to be used either as Ground Control Points (GCPs) for georeferencing or as independent Check Points (CPs) during tests is presented.

### Experimental Set-Up

**Figure 3:**
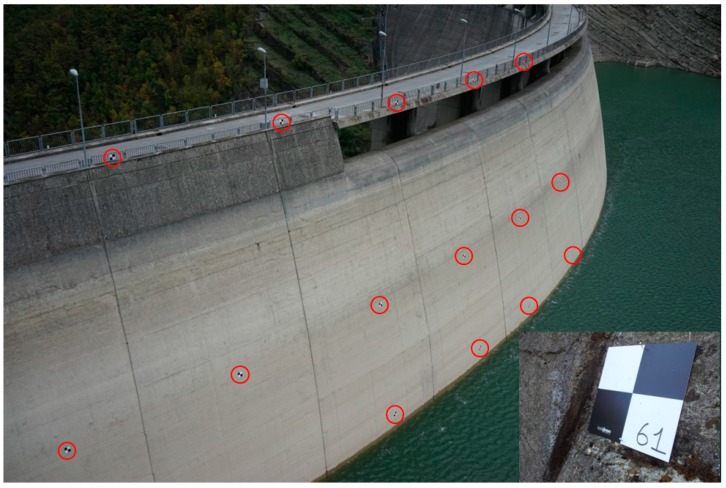
Upstream face of the Ridracoli dam. The red circles indicate marker positions. The small panel shows a marker applied on the structure.

The aerial platform is a HighOne 4HSE Pro quadrotor (Italdrone, Ravenna, Italy) mounting a gimbal system and an Alpha 7R, 36.4 Mpix Full frame camera (Sony) oriented with its axis along the perpendicular (Figure 2). The gimbal compensates drone vibrations due to the wind and to the flight operations. The lens is a 35 mm f/9 and the Sony Alpha 7 camera has the AF—Autofocus—Lock feature that manages the framing dimensions in relation to the subject characteristics and also allows an auto-focus procedure. The camera focus, after being tested on a portion of the structure, was set to infinity during the survey. The dam, the ancillary structures and the surrounding land were inspected by drone. Around 200 images of the upstream face were shot. To georeference the frames, before the drone survey, a set of markers were deployed on the dam structure to be used as Ground Control Points. Each marker has a squared shape and is 0.40 m high (Figure 3). 

**Figure 4:**
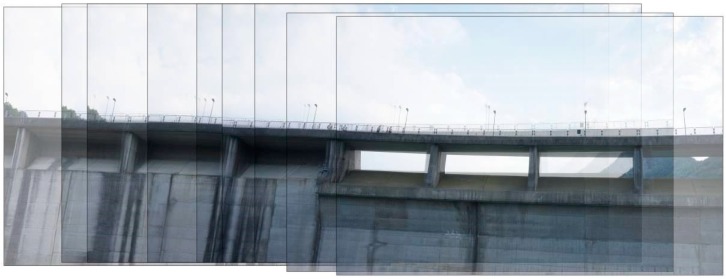
Images overlap by more than 70%.

In two different moments of the year, sixty regularly-spaced markers were placed on three rows on the upstream face (Figure 3). In August 2015, two rows of markers were applied on the crest railing and on the upstream surface at the hydrostatic level of the time (i.e., 543.28 m a.s.l.) using a boat. While in October 2015 a third row of markers was deployed at the new hydrostatic level (i.e., 533.65 m a.s.l.). The UAV survey of the upstream face was performed after this last marker deployment to ensure uniform shooting conditions of the frames [65]. The image size is 7360 × 4912 pixels with a resolution of 350 dpi. The images were shot at a distance of about 15 m from the dam; the Ground Sample Distance (GSD) equals 2.1 mm. The images are shot every 1.87 s and the images overlap by more than 70%. An example is provided in Figure 4.

**Figure 5:**
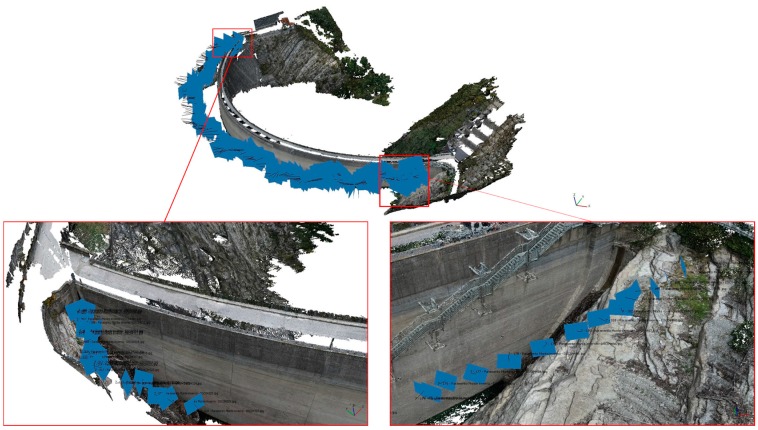
Camera positions (upper panel) and two enlargements for the left and right abutments (lower left and right panels, respectively).

The camera positions are represented in Figure 5; because of the great extent of the structure an enlargement of the two boundaries is provided.

We recall that this paper aims at evaluating the influence of both GCPs layout and number on the accuracy of a 3D dense point cloud of the structure. Moreover, we want to determine which is the effect of the GCPs location choice on the dense point cloud at the boundaries of the structures, i.e., at both the right and the left abutments. To this end, a high number of markers (i.e., 60) were placed on the dam upstream face. To analyze the effects of marker location, 25 different GCPs layouts were chosen. In each layout, markers are used either as GCPs to georeference the frames or as CPs to test the resulting model accuracy. The former are represented as black circles, the latter as empty circles (Table 1). Marker configurations are grouped according to the GPCs density on a 9 × 9 square marker formation. This representation is useful for analyzing the effects of GCPs location, the density being equal. For instance, to evaluate the effects of different marker configurations, the pattern being equal, the following three groups are investigated: *b*, *g* and *j*; *a*, *d* and *r*; *f*, *n* and *y*. To evaluate the effect of deploying GCPs along one row at different elevations, the layouts *a*, *d*, *e*, *l*, *f*, *p*, *r* and *y* are analyzed. For instance, in layouts *a*, *d* and *r*, GCPs are placed only on the dam crest (i.e., the first row) with consistently different spacing. In a similar way, in layouts *f* and *y* GCPs are placed on the upper and lower rows with variable density. 

To evaluate how the GPCs location effects the dense point clouds at the boundaries, we chose configurations that are similar but with different GCPs numbers and positions at the abutments. This is the case of the two groups of configurations *c* and *e* and *i* and *n*. Each couple has the same GCPs density, however, only one of the two supplies markers at the dam boundaries. It is worth underlining that also configurations with a very low number of GCPs were chosen to investigate the widest range possible of GCPs configurations. 

**Figure 6:**
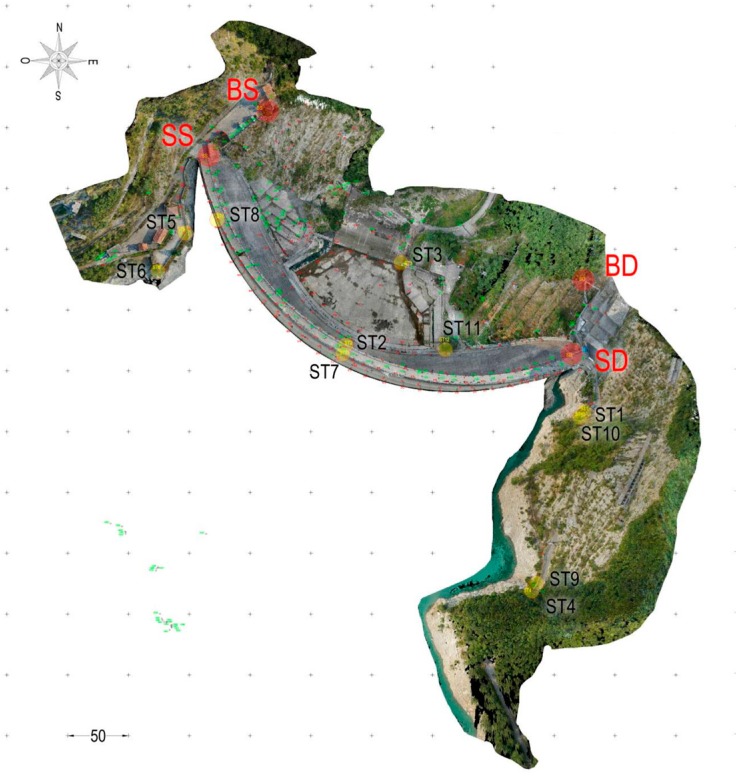
Orthophoto of the dam and the materialization vertices of the two geodetic networks used for the topographic survey. The vertices of the existing network materialized by little pillars are red, the vertices of a new network materialized by topographic nails fixed on the ground are yellow.

The coordinates of the markers were acquired through a traditional survey technique using a Total Station TS30 (Leica-Geosystems). To this end, a pre-existing geodetic network was used for monitoring purposes. The existing network consists of four vertices materialized by little pillars (i.e., BS, SS, DS and BD, Figure 6). Besides the existing network, a new one was setup with 7 vertices materialized by topographic nails fixed on the ground (Figure 6). The new network was connected to the first one through topographic triangulation measurements.

To improve the accuracy of the measured coordinates, a hyper-deterministic scheme was adopted and the acquired observations were treated rigorously, executing least-squares compensation of topographic measurements. The standard deviation of each point is lower than 1 cm along the three directions. The mean value of all standard deviations equals 1.0 cm, 1.0 cm and 0.8 cm along the three directions, respectively. Due to the low error values, the points are suitable for either georeferencing the images or for validating the model. For more details about the traditional topographic survey, the reader can refer to the work of Buffi et al. [65].

## 3. Image Error Analysis

**Figure 7:**
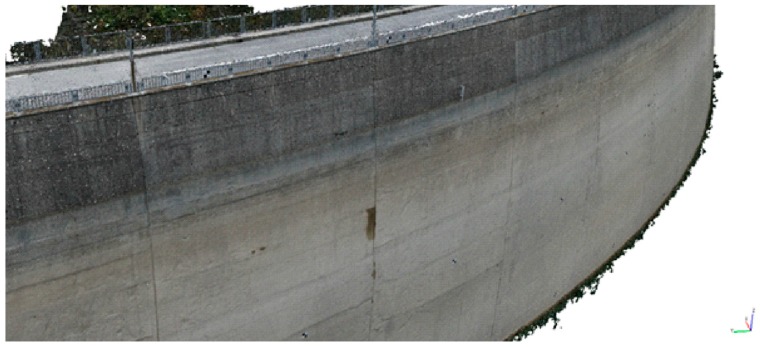
UAV dense 3D point cloud of a portion of the upstream face of the Ridracoli dam.

The frames acquired by UAV are used to build a 3D dense point cloud model of the upstream face through the Structure from Motion (SfM) technique (Figure 7). The SfM technique allows the construction of a three-dimensional dense point cloud model of an object starting from the automatic collimation of frames [33]. At first, the procedure consists in performing a feature point detection and matching, using automatic algorithms. Then, incrementally, this procedure adds images, triangulates matching features and refines the scene using bundle adjustment, allowing for the construction of the 3D point cloud. The Agisoft Photoscan^®^ (vers. 1.2.4) software was employed to build the 3D dense point cloud. To derive the dense point cloud, the procedure is presented in the study by Jaud et al. [66].

First, the frames are uploaded. On photographs, common tie points are found and matched. The external camera orientation parameters are detected for each image. The Brown’s distortion model is used to simulate the lens distortion, and camera calibration parameters are found. This model allows for correcting both radial and tangential distortion. The coordinates of the markers used as GCPs are associated to the corresponding marker centre. This procedure has the purpose of georeferencing the frames on which the markers are used to build the most accurate possible dense point cloud. Secondly, the dense point cloud is built using the camera positions and the images and the CPs coordinates are extracted from the dense point cloud. The camera model has been optimized after the photos aligning. The parameters of the camera model are shown in Table 2.

The model is built using the Photoscan setting values reported in Table 3. Concerning image coordinates accuracy, the tie point accuracy equals 1 pixel, marker accuracy equals 0.1 pixel. Agisoft Photoscan^®^ (ver. 1.2.4) provides an average RMS error for tie points equal to 0.798 pixel and an RMS average projection error equal to 1.216 pixel (Table 3).

It is possible to assess the errors between the coordinates of the CPs on the dense point cloud and compare their actual coordinates measured by total station. The accuracy of the dense point cloud model depends on the number and configuration of the set of GCPs used to georeference the frames. The CPs coordinates extracted from the dense point cloud are hereafter referred to as ‘extracted coordinates’. The accuracy analysis is performed in accordance with geospatial positioning accuracy standards [67]. For each layout, the errors along the elevation (i.e., *ε_z_*), the North and East directions (i.e., *ε_Y_* and *ε_X_* respectively) are evaluated for each CP, comparing measured and extracted coordinate values:

(1) $$\varepsilon_{z}=Z_{obs}-Z_{estimated}$$

(2) $$\varepsilon_{Y}=Y_{obs}-Y_{estimated}$$

(3) $$\varepsilon_{X}=X_{obs}-X_{estimated}$$

Moreover, the error vector lying on the North-East plane (i.e., *ε_XY_*) is evaluated for each CP:

(4) $$\varepsilon_{XY}=\sqrt{\varepsilon_{X}{}^{2}+\varepsilon_{Y}{}^{2}}$$

To estimate the overall quality of each GCPs layout, the Mean Absolute Error (MAE) is evaluated as follows:

(5) $$MAE_{j}=\frac{1}{N}\sum_{i=1}^{N}|\varepsilon_{j,i}|$$

where *ε_j_*_,*i*_ is the error along the *j*-th direction (i.e., North, East, elevation and on the North-East plane, therefore *j* = 1, …, 4) of the *i*-th check point measured by the topographic survey and *M* is the number of check points for each specific layout. The Mean Absolute Error measures the overall match between observed and simulated coordinate values. A perfect GCPs layout would result in an MAE equal to zero. This metric estimation does not provide any information about under- or over-estimation, but it determines all deviations from the observed values regardless of the sign.

## 4. Results and Discussion

**Figure 8:**
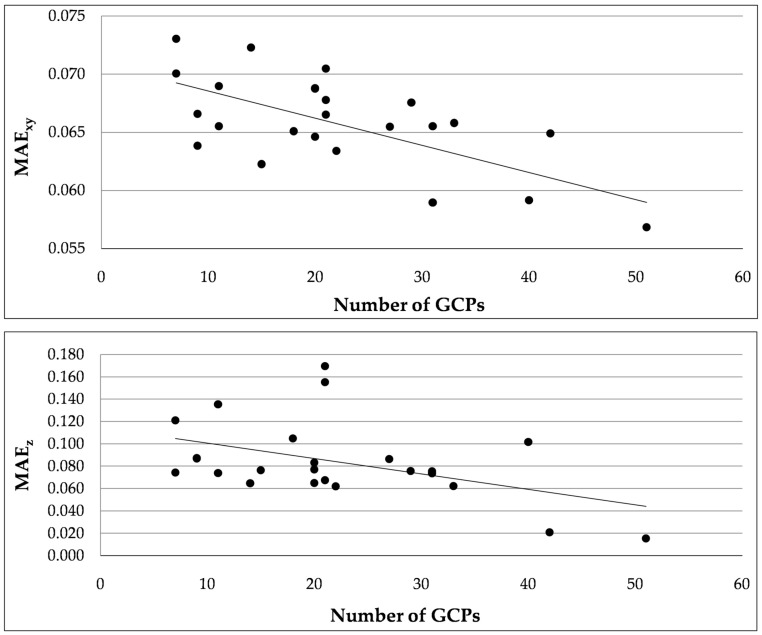
Mean Absolute Error (MAE) on the North-East plane and along the elevation (i.e., z direction) against the number of Ground Control Points (GCPs).

To analyze the effect of GCPs layout on the dense point cloud accuracy, the errors between measured and extracted coordinates of CPs are estimated for each layout. As expected, both in the altitude direction and on the North-East plane the MAE value decreases when the number of GCPs increases (Figure 8).

However, it is possible to observe that configurations with the same number of GCPs can have a consistently different MAE value. For instance, this is the case of the configurations *q*, *s* and *t*. They are all characterized by 31 GCPs, but at the same time by a consistently different MAE in the altitude direction and on the North-East plane, as also shown in Table 4. It is interesting to note that they have the same number of GCPs but different density as their pattern is different. Configurations *j*, *l* and *r* consist of 21 GCPs, but are characterized by different error values in all directions. Layouts with a similar pattern (e.g., configurations *f* and *y*) can be characterized by different error values along the elevation. This is due to the fact that it is the combination of both GCPs density and pattern that defines the accuracy of the dense point cloud. Both density and layout are useful as they can consistently speed up the operations for the experimental setting.

**Figure 9:**
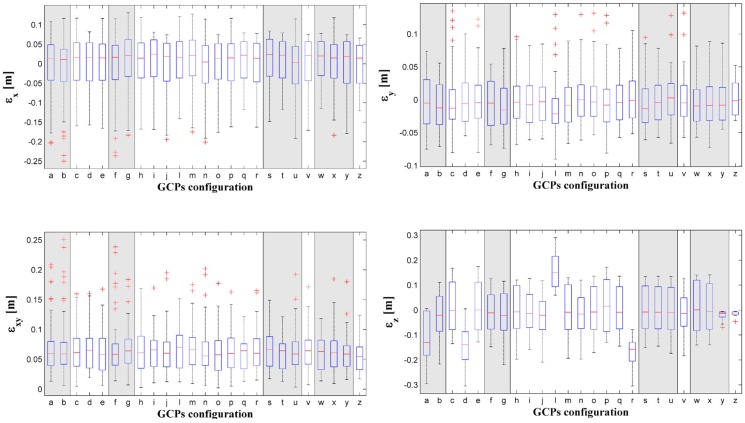
Boxplots of errors (*ε*) along North, East, elevation (y, x and z) directions and on the North-East plane for each GCPs layout. The background colour groups layout with the same density as in Table 1.

The boxplots of errors in the three directions North, East and Elevation and on the North-East plane are reported in Figure 9. The boxplots visually represent the median, the second and third quartiles of errors for each GCPs combination. Moreover, the boxplots highlight the presence of outliers, which are those CPs with higher error value than any other CPs. Along the elevation, the marker layout with the smallest MAE is the *z*, followed by *y* as also shown in Table 4. The former layout is characterized by the highest number of GCPs (i.e., 51), and GCPs are deployed in a way that ensures the almost complete coverage of the dam upstream face. Indeed, in all rows except in the intermediate one, the GCPs are placed in every position. 

**Figure 10:**
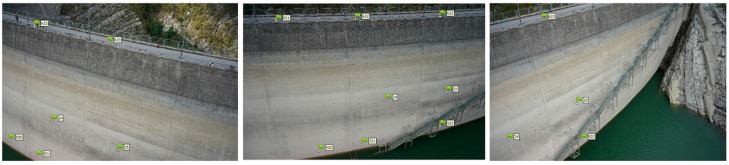
An example of the influence of the percentage of water on the RMS image value: from left to right, the RMS error of the image increases with the percentage of water. In the images, the green flags represent the markers.

It is worth underlining that the configuration *y* has the second lowest MAE value, even though the second row in not secured with any GCP. This is due to the fact that the first and the lowest rows are responsible for the accuracy of the model if not provided by markers as they are the most difficult to capture during the UAV survey, for two different reasons. Indeed, the last row is close to the lake level, therefore, the UAV cannot move close to the markers and cannot shoot images from every angle, while the low accuracy of the first row is due to the fact that the first row of markers is applied to the balustrade on the dam crest. The frames shot of the balustrade capture the empty spaces in between the balustrade sticks. It results in overexposed frames because of the automatic brightness adjustment of the camera shutter and are then characterized by a low quality. The RMS error of the images that constitute the dense point cloud close to the spillway and to the dam crowning is higher than the other images because it includes the sky and/or water. An example is provided by Figure 10. The images show the same part of the dam. From left to right, the greater the quantity of water in the images, the higher the RMS error of the image. In fact, the RMS errors are, respectively, 0.975, 1.011 and 1.368 pixel. To improve the accuracy of the georeferenced dense point cloud, it is important to deploy a high number of equally-spaced GCPs on the first row. Configurations with a low number of GCPs on the balustrade (i.e., the first row on the upstream face) have high error values. 

**Figure 11:**
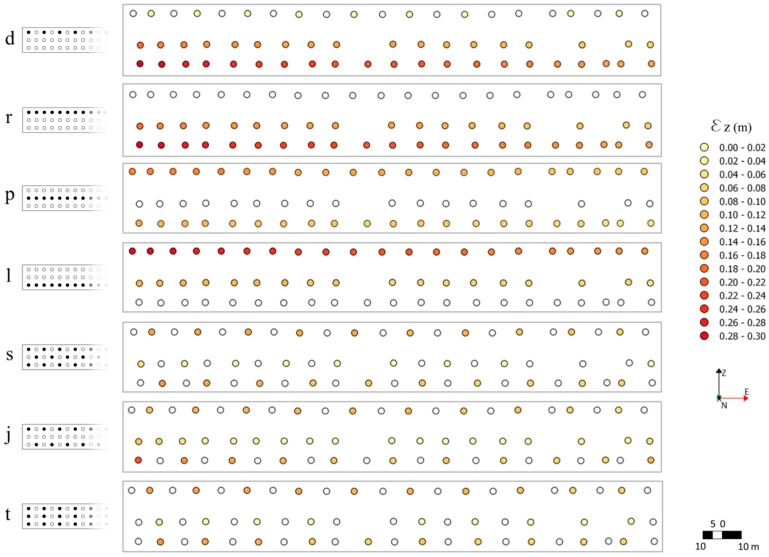
Magnitude of error along the elevation (*ε*_z_) for each marker, considering seven different marker configurations.

The error values along the altitude prove the influence of GCPs pattern (Figure 11). If the GCPs are deployed on the crest, as in combinations *d* and *r*, the crest is characterized by the lowest error values, while the highest error values are concentrated on the lowest row, at the bottom of the upstream face. Markers on the lowest row are difficult to survey because of the proximity to the water. In this case, the MAE value is more conditioned by the GCPs pattern than by their number. A similar behavior can be observed in combinations *l* and *p*, where the GCPs are deployed either on the middle row or on the lowest row, respectively. 

In both cases, the highest error values are concentrated on the first row as the balustrade effects the frames quality, as mentioned before. It is interesting to note that when the markers are placed on both the upper and lower rows, the errors decrease consistently, as for combinations *s*, *j*, *t*. The combination pattern and number of GCPs defines the accuracy of the resulting 3D model. As a matter of fact, configurations *r*, *l* and *j* are characterized by the same number of GCPs (i.e., 21), however, their different layouts result in very different error values. It is worth noting that the error along the elevation in percentage is very small when compared to the dam height. The minimum error value is 0.015% of the dam height (i.e., 103.5 m). Indeed, the error along the elevation of configuration *z* is 1.5 cm. Configurations *z*, *y*, *i*, *v*, *f*, *n* and *j* are all characterized by an MAE_z_ (see Table 4) equal to 0.015 m, 0.021 m, 0.062 m, 0.062 m, 0.065 m, 0.065 m and 0.066 m, respectively, and thus lower than or equal to 0.067% of the dam height. Thus, these 3D models seem to be characterized by error values that are compatible with a Finite Element analysis. Indeed, for a FE analysis, an error of 0.1% of the dam height is acceptable, in this case equal to 0.1 m [62]. It is important to point out that the model of the spillways must be accurate. Indeed, an inaccurate representation of the spillway geometry can cause an inaccurate representation of the overflowing water. Taking into account an error on the evaluation of the overflow equal to the aforementioned 0.067% of the dam height, the consequent error on the peak discharge would be around 4.5% of the design peak. This error is compatible with the uncertainties in the flood peak estimation [63].

The MAE values on the North-East plane lead to the same conclusions (Figure 9, lower left panel). Indeed, the configuration *z* has the lowest value in this direction, as it does along the elevation. It is followed by the configuration *t*, in which the GCPs are placed on the three rows in every other position, ensuring a good coverage of all the upstream face. 

It is interesting to highlight that the best performing configurations are in agreement with the recommendations of literature. Indeed, GCPs are widely distributed across the target area [54] and at the edge of the upstream face [55]. Results suggest that GCPs distribution needs to be adapted to the object surveyed and to the distance of the UAV from the observed object, as in Harwin and Lucieer [60]. In our work, we found that the “threshold distance” between the GCPs (distance beyond which results do not improve) is 13 m. This distance is greater than that recommended by Harwin and Lucieer [60]. A minimum spacing of 13 m has shown a good performance both for a drone survey and for a classic laser scanning survey, as confirmed by Buffi et al. [68].

### The Dense Point Cloud Accuracy at the Dam Boundaries

**Figure 12:**
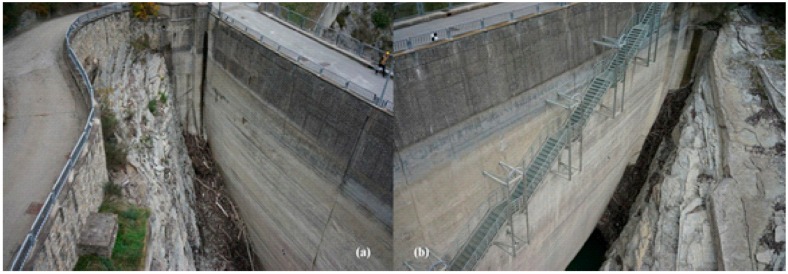
(**a**) Left rock abutment and (**b**) right rock abutment and the stairway element on the upstream face.

The boundaries of the dam are among the most important parts of the dam since they define the connections between the structure and the surrounding terrain. However, because of the shape of both right and left abutments, dam boundaries are not easily accessible by drone survey (Figure 12a,b). 

**Figure 13:**
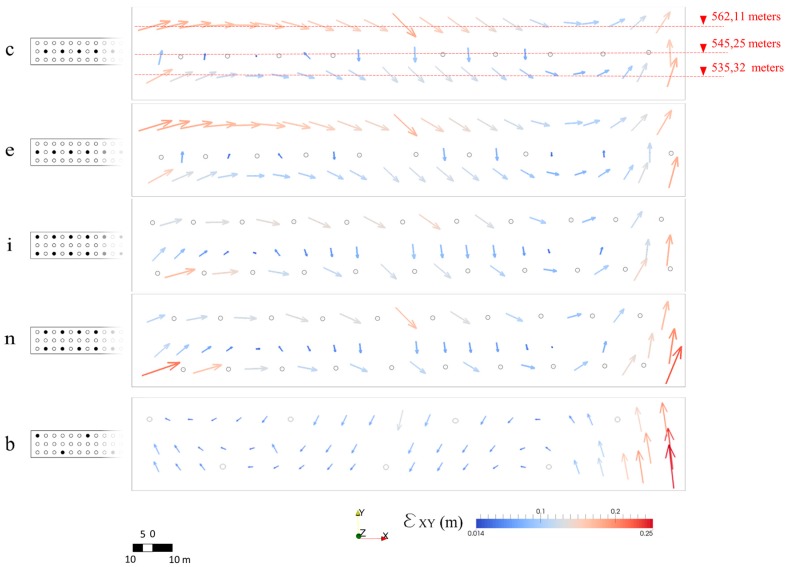
Error along the North-East plane (*ε*_XY_) for each CP for GCPs configurations *c*, *e*, *i*, *n*, *b*. The placement of the arrows (indicating the error magnitude and direction) and of the grey circle is in accordance with the location of the GCPs—positioned at different levels on the dam upstream face—to facilitate the reading of the data. In particular, the figure on the top shows the altitude (meters above sea level) of the location of the GCPs.

Thus, images shot of the boundaries are characterized by perspective distortion. Moreover, the presence of the stairways on the right side made it difficult to survey that portion of the dam. To increase the accuracy of the resulting dense point cloud it was necessary to place markers in the proximity of the rocks and under the stairways on the right side of the structure. The high number of frames and the presence of GCPs made it possible to increase the accuracy of the dense point cloud as is evident from the performance of configuration *z*. To deeply investigate the effects of the GCPs pattern at the boundaries, the two configuration couples *c*, *e* and *i*, *n* are analyzed. Configurations *c* and *e* both have the same GCPs density and the same pattern. However, GCPs are placed in the proximity of the boundaries only in configuration *c*. This results in high error values at the boundaries, as testified by the number of outliers along the north direction (Figure 9). Layout *c* leads to high error values at the left and right boundaries because of the absence of the GCPs (Figure 13). The error is higher at the right side where the presence of the stairways makes it difficult to capture the dam face. Layout *e* differs from *c* as markers are placed at both sides of the upstream face. However, both configurations are characterized by high error values on the dam crest. It is worth noting that the spillway gates are characterized by a high error value as the openings imply an uncertain dense point cloud when no markers are placed in the proximity. Similarly, configuration *b* is effected by the absence of GCPs in the proximity of the right abutment where the presence of a stairway and of rocks makes it difficult to capture the dam surface on the North-East plane (Figure 13). The effect on the boundaries is more evident comparing layout *i* with layouts *n* and *b* (Figure 13). GCPs are placed at both sides of the upstream face only in configuration *i*. This leads to a high error value at the boundaries for configuration *n* and *b*, while the error is lower for configuration *i* at the same location.

Moreover, it is worth noting that all configurations except *b* in Figure 13 present a systematic error in areas characterized by a low GCPs density. A more uniform distribution of the GCPs across the dam upstream face would substantially mitigate the systematic errors. This finding is in accordance with James et al. [69] who noticed this behavior while estimating the accuracy of DEMs georeferenced with different sets of GCPs.

## 5. Conclusions

This work explores the effects of ground control point position and number on the accuracy of a dam dense point cloud, obtained by a drone survey. As GCP deployment is a time and money-consuming task, especially on large structures, this paper aims to provide principles for supporting the GCP deployment on high-rise buildings in order to speed up operations on site. 

As expected, the results show that the model performs better when the density of markers is high. However, it is the combination of both GCP pattern and GCP density that determines the gain in accuracy. Results highlight that the error values show a higher variability along the elevation than along any other direction because of the high-rise characteristics of the dam. Therefore, GCPs should be placed at different elevations to increase the accuracy of the resulting dense point cloud. Moreover, where the structure is characterized by discontinuities such as spillway gates, it is necessary to place GCPs in the proximity of the openings to gain in accuracy. In addition, the presence of a balustrade, water, sky and uniform texture increases the RMS error of the images. In order to increase the accuracy of the georeferenced model special attention should be paid to the marker placement, in particular near the spillways, balustrade and hydrostatic level. 

This paper also draws attention to the dam boundaries, where the presence of rocks reduces the accessibility to the dam and reduces the image quality. Since modeling the connection between the structure and the surrounding terrain is a relevant issue, it is necessary to deploy GCPs in the proximity of the abutments, avoiding patterns without GCPs at the boundaries. Moreover, we noticed that it is important to shoot a high number of images from either side of a singularity. For instance, this is the case of the right abutment, which was captured by a high number of shots from left to right and by a small number from right to left. In this case, the accuracy can be substantially lower, especially if a small number of markers are placed at the boundary.

These findings can be extended to other high-rise structures. For instance, if the façade of a structure is characterized by openings, as in the case of the spillways, it is necessary to deploy GCPs in the direct proximity of these openings to increase the accuracy of the photogrammetric survey. 

In accordance with literature guidelines, GCPs should be widely distributed across the target area and at the edge of the upstream face. To reduce the occurrence of systematic errors, GCPs should be better distributed across the dam upstream face. Future developments could include an analysis of any changes in the accuracy of the model obtained by varying the overlapping of the frames or removing poor quality images.

## Figures and Tables

**Table 1:** Marker layouts with IDs ranging from a to z. The black circles are the markers used as GCPs, the real coordinates of which are used to georeference the dense point cloud, while the empty circles are the markers used as control points (CPs). The coordinates of CPs are extracted from the model and compared with their actual coordinates to evaluate the accuracy of the model.

Density	Layout					
1/9	a	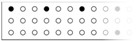	b	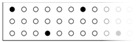		
1/6	c	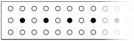	d	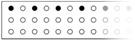	e	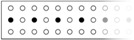
2/9	f	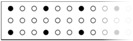	g	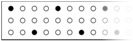		
1/3	h	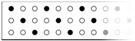	i	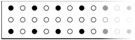	j	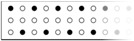
	l	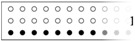	m	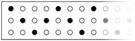	n	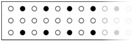
	o	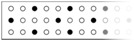	p	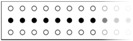		
	q	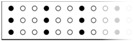	r	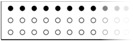		
1/2	s	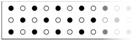	t	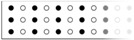	u	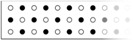
5/9	v	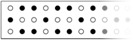				
2/3	w	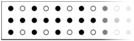	x	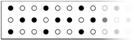	y	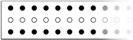
5/6	z	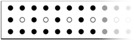				

**Table 2:** Parameters of the camera model.

Focal lenght (pix)	fx	7424.32
	fy	7428.37
Principal point offset (pix)	cx	3654.48
	cy	2434.65
Skew coefficient (pix)	skew	−5.89
Radial distortion coefficient	k1	0.05
	k2	−0.25
	k3	0.04
Tangential distortion coefficent	p1	0.00
	p2	0.00

**Table 3:** Workflow and parameters used as Photoscan^®^ input to build the dense point cloud.

Workflow	
**Align Photo**	
Accuracy	Medium
Pair pre-selection	Disabled
Point Limit	40,000
**Build Preliminary Mesh**	
Surface type	Arbitrary
Source data	Sparse
Interpolation	Enabled
Polygon count	Custom
Point classes	All
**Import GCPs (GCPs Settings)**	
Camera accuracy (m)	10
Marker accuracy (m)	0.005
Tie point accuracy (pix)	1
**Build Dense Cloud**	
Quality	Medium
Depth filtering	Aggressive

**Table 4:** Ground Control Points (GCPs) ID for each layout grouped by density, corresponding number of GCPs for each layout, Mean Absolute Error values (MAE) for the North, East, elevation (z) directions and the MAE evaluated on the North-East plane for each marker layout.

Density	Combination ID	Number of GCP	MAE_x	MAE_y	MAE_xy	MAE_z
		-	m	m	m	m
1/9	a	7	0.058	0.032	0.070	0.121
	b	7	0.059	0.032	0.073	0.074
1/6	c	9	0.053	0.033	0.067	0.087
	d	11	0.054	0.031	0.066	0.135
	e	9	0.052	0.031	0.064	0.087
2/9	f	14	0.061	0.030	0.072	0.065
	g	11	0.056	0.032	0.069	0.074
1/3	h	20	0.053	0.030	0.065	0.077
	i	22	0.053	0.029	0.063	0.062
	j	21	0.054	0.029	0.066	0.066
	l	21	0.053	0.037	0.068	0.155
	m	20	0.055	0.032	0.069	0.083
	n	20	0.056	0.030	0.069	0.065
	o	15	0.051	0.029	0.062	0.076
	p	18	0.052	0.033	0.065	0.105
	q	31	0.049	0.027	0.059	0.073
	r	21	0.056	0.030	0.067	0.170
1/2	s	31	0.054	0.031	0.066	0.075
	t	31	0.049	0.027	0.059	0.073
	u	29	0.055	0.030	0.068	0.076
5/9	v	33	0.053	0.031	0.066	0.062
2/3	w	40	0.048	0.029	0.059	0.102
	x	27	0.054	0.029	0.065	0.086
	y	42	0.055	0.027	0.065	0.021
5/6	z	51	0.050	0.023	0.057	0.015
